# Conditioned Media from Mechanically Stimulated Macrophages Upregulate Osteogenic Genes in Human Mesenchymal Stromal Cells

**DOI:** 10.1002/adhm.202500706

**Published:** 2025-06-22

**Authors:** Anne Géraldine Guex, Ursula Menzel, Yann Ladner, Angela R. Armiento, Martin J. Stoddart

**Affiliations:** ^1^ AO Research Institute Davos Davos 7270 Switzerland; ^2^ Department Research University Center for Dental Medicine Basel UZB University of Basel Basel 4058 Switzerland; ^3^ Department of Biomedicine University of Basel Basel 4031 Switzerland; ^4^ Institute for Biomechanics ETH Zurich Zurich 8008 Switzerland; ^5^ Department of Orthopedics and Trauma Surgery Medical Center‐Albert‐Ludwigs‐University of Freiburg Faculty of Medicine 79110 Freiburg Germany

**Keywords:** bone fracture healing, compression and shear, fibrin, macrophage polarization, mechanical load, mesenchymal stromal cells

## Abstract

Bone healing is a multifaceted scenario with tightly orchestrated sequences that decide upon successful bone fracture healing or nonunion. In this context, the immune system, particularly the impact of macrophages on mesenchymal stromal cell (MSC) recruitment and differentiation, cannot be overemphasized. Further adding to the complexity, fracture healing is not only governed by chemical signals but strongly depends on mechanical stimulation. Here, an in‐house built bioreactor is used to culture THP‐1 macrophages, stimulated with lipopolysaccharide and interferon‐gamma (M(LPS)) or interleukin 4 (M(IL‐4)) in fibrin hydrogels under compression and shear. Subsequently, MSC‐pellets are cultured in conditioned media, derived from macrophages, and analyzed for chondrogenic or osteogenic gene expression after 9 days. In M(IL‐4) conditions under mechanical load, expression of *IL1B, IL6, TNF, IL10, CCL18, CD163*, and *CD206* is increased compared to the static condition. In MSCs, osteogenic genes *RUNX2* and *ALPL* as well as chondrogenic genes *ACAN* and *COL2A1* are increased in conditions treated with a medium derived from mechanically stimulated macrophages. The results suggest that culture in fibrin and under loading induces a complex macrophage polarization phenotype which affects processes during endochondral ossification in human MSC.

## Graphical abstract

Bone fracture healing is a complex, timely orchestrated scenario involving multiple cell types and a plethora of cytokines and regulatory factors. To gain further insight, an in vitro model to a) study macrophage polarization under mechanical load in a fibrin hydrogel and b) subsequently assess the effect of conditioned media derived from macrophages on mesenchymal stromal cell differentiation, is developed.

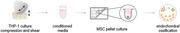

## 1 Introduction

Bone fracture healing is a complex process with tightly regulated and interdependent sequential phases, involving multiple cell types and a plethora of cytokines.^[^ ^1^, ^2^ ^]^ At the onset of the healing cascade lie immune cells, in particular macrophages.^[^ ^3^, ^4^, ^5^ ^]^ In response to bone fracture, macrophages enter the site of injury and act as key mediators to initiate, maintain, and resolve an inflammatory response. Their regenerative capacity ranges from tissue cleavage to secretory factors that attract progenitor cells which ultimately form the new tissue. Seconds after fracture, fibrinogen and thrombin‐rich peripheral blood enter the fracture site and form a highly crosslinked fibrin network to which macrophages invade first, subsequently attracting progenitor cells.^[^ ^3^ ^]^ Fibrin therefore forms the first substrate for invading cells to proliferate and differentiate. This 3D environment, rich in soluble factors and architectural cues, is instructive for macrophage polarization and facilitates migration. Locally derived progenitors from the bone marrow or the periosteum are key players to be recruited to the site of injury and promote endochondral ossification.^[^ ^1^ ^]^ The crosstalk between macrophages and progenitor cells has been appreciated as vital in this process, with macrophage depletion causing a reduction in callus size, reduced chondrogenesis and ultimately leading to impaired fracture healing.^[^ ^3^, ^4^, ^6^ ^]^

In addition to soluble and biophysical factors, cells in the fracture gap are subjected to mechanical stimuli in the form of shear flow, compression, strain, or tension which are essential for progenitor cell differentiation.^[^ ^7^ ^]^ Under rigid fixations, fractures heal following the concept of primary bone formation with direct osteogenesis of mesenchymal stromal cells (MSC). Subjected to micromotion, bone heals in a concept defined as endochondral ossification, via a callus and cartilaginous template.^[^ ^8^ ^]^ How mechanical load governs these distinct processes and how it is converted to biological signals is poorly understood. Intuitively, cells of the musculoskeletal apparatus (bone, muscle, tendon, or cartilage) are the most responsive to mechanical stimuli, but growing evidence suggests a major effect of mechanical stimuli on macrophage polarization and their pro‐ or anti‐inflammatory phenotype.^[^ ^9^ ^]^ Research data from spaceflights and experiments in low gravity underpin the importance of mechanical stimulation for the majority of cells in the human body, including immune cells.^[^ ^10^, ^11^ ^]^ Conversely, overload or excessive mechanical stimulation has been associated with delayed healing or non‐union, increased secretion of pro‐inflammatory factors, fibrosis, cartilage damage, or osteoclast activation.^[^ ^12^, ^13^, ^14^, ^15^, ^16^, ^17^ ^]^ In vitro, rat fibroblast‐like synoviocytes subjected to 10% stretch at 1.0 Hz on a Flexcell FX5000 Tension System were shown to undergo activation of the Piezo1/NF‐κB/NLRP3 pathway resulting in increased expression of, e.g., interleukin 6 (IL‐6) or tumor necrosis factor alpha (TNF‐α) compared to the static control, indicative for an inflammatory environment related to osteoarthritis.^[^ ^18^ ^]^ Further, delayed loading (4 days post‐fracture) of 0.5 N in mice resulted in enhanced and stronger callus formation compared to 1 or 2 N loading.^[^ ^19^ ^]^ In the context of the immune response during bone fracture healing, scientific evidence from systematically conducted in vitro studies is rare, yet mechanical stimulation is suggested to accelerate a timely transition from a pro‐inflammatory phenotype (also referred to as M1), to an anti‐inflammatory or alternatively activated phenotype (also referred to as M2). This, in certain experimental settings, was shown to improve migration and osteogenesis of progenitor cells.^[^ ^20^, ^21^ ^]^ While classically activated, pro‐inflammatory macrophages are characterized by secretion of interleukin 1 beta (IL‐1β), IL‐6, and TNF‐α, anti‐inflammatory macrophages secrete transforming growth factor beta (TGFβ), vascular endothelial growth factor (VEGF), or interleukin 10 (IL‐10) – among other cytokines. Pro‐inflammatory cytokines have been associated with reduced osteogenesis, increased osteoclast activation, and cartilaginous matrix degeneration, while anti‐inflammatory cytokines have been shown to stimulate osteogenesis and reduce osteoclast activity.

For successful fracture healing and bone union, these factors have to act in a time‐ and concentration‐controlled manner. Identifying patterns and understanding these interactions in predictive in vitro models will provide fundamental knowledge on bone fracture healing with the possibility to develop immunomodulatory biomaterials for non‐union fractures and suggest physiotherapeutic regimes or micro‐movements to accelerate healing. In vitro, cell experiments on 2D culture substrates and under static conditions not only remove cells from their natural 3D environment but also deprive them of mechanical stimuli which are vital for homeostasis and physiology. Consequently, first‐line in vitro evaluations fail to elucidate the role of macrophages during bone fracture healing and have limited predictive power. To account for this, bioreactors were developed to culture MSC, chondrocytes, osteogenic progenitors, osteochondral plugs, or intervertebral discs under load,^[^ ^22^, ^23^, ^24^ ^]^ with fewer studies focusing on the culture of macrophages under load.^[^ ^9^, ^21^ ^]^ In recent years, however, scientific evidence underpinned the importance of addressing the immunomodulatory effect of mechanical stimulation on macrophages by use of bioreactors to apply strain,^[^ ^25^ ^]^ hydrostatic pressure^[^ ^26^ ^]^ or compression and shear.^[^ ^27^ ^]^

In this work, we established a bi‐phasic in vitro model that first recapitulates the mechanically dynamic, 3D environment of macrophages in the callus, and second mimics the regenerative potential of macrophage secretomes on progenitor cells. An in‐house designed multiwell bioreactor was used to subject macrophages (THP‐1) to compressive load and shear. To mimic the haematoma‐like 3D environment of a freshly formed callus upon bone fracture, THP‐1 cells were cultured in a fibrin hydrogel. Collected conditioned medium was then added to human bone marrow‐derived MSC pellet cultures, which were assessed for the expression of chondrogenic or osteogenic genes, or genes encoding for inflammatory proteins or matrix‐degrading enzymes.

In summary, the immune response, comprising the secretion of pro‐inflammatory and anti‐inflammatory factors is essential to initiate healing and stimulate tissue remodelling.^[^ ^5^ ^]^ However, it has not been elucidated how the secretion of factors from mechanically stimulated macrophages in vitro influences endochondral ossification during early time points in an in vitro model. This study aims to advance research on the crosstalk between immune cells and osteogenesis and seeks to understand the influence of mechanical stimulation and 3D environments on macrophage polarization and downstream impacts on MSC differentiation.

## 2 Results and Discussion

### 2.1 Mechanical Stimulation Induces a Distinct Phenotype in THP‐1 Macrophages

**Scheme 1:**
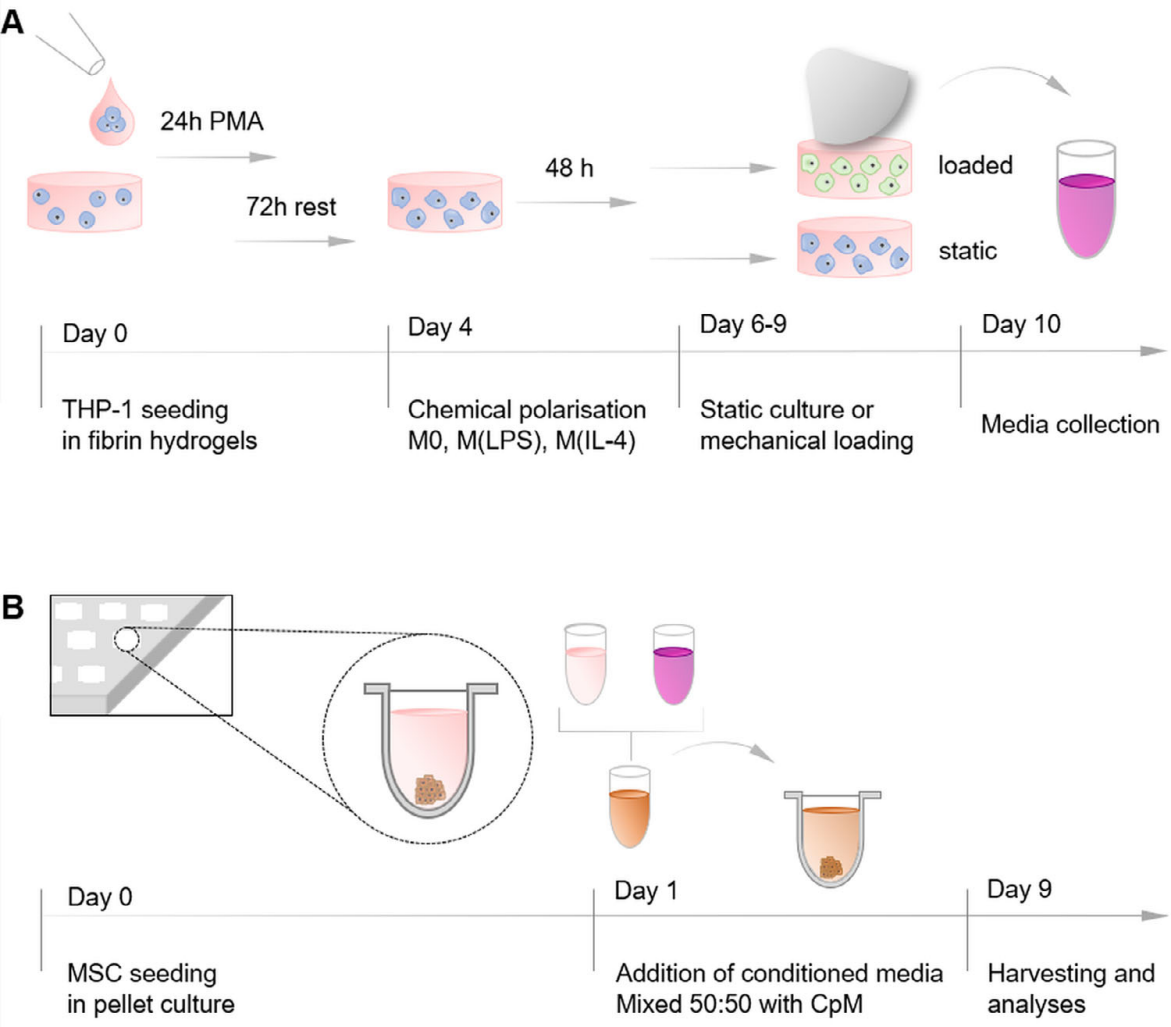
A) THP‐1 cells were seeded in fibrin hydrogels. The monocyte‐to‐macrophage transition was induced with PMA, followed by a 72‐h rest period. Following, macrophage polarization was chemically induced with lipopolysaccharide and interferon‐gamma (M(LPS)) or interleukin 4 (M(IL‐4)). After 48 h, cell‐fibrin constructs were either cultured under static conditions or mechanically loaded for 1 h a day over 3 days. Conditioned media were collected and cell‐fibrin constructs were harvested for further analyses. B) MSC pellets were prepared in a v‐shaped 96‐well plate. After 24 h, conditioned media from macrophages were added. MSC pellet culture was maintained for 9 days.

Since the 19th century, according to Wolff's law,^[^ ^28^ ^]^ it has been a well‐accepted concept, that bone adapts to the mechanical load it is subjected. Conversely, bone that is not loaded will degenerate over time, as observed in multiple clinical scenarios. Despite general agreement on the importance of mimicking the native environment and major advances in bioreactor and biomaterials design, a large proportion of cell culture experiments are still conducted under static conditions and on 2D culture substrates. The importance of mechanical stimuli on immune cells, and in particular macrophages, was long overlooked and has only recently appeared in the spotlight of modern research. Increasing evidence suggests that macrophages also adapt and change phenotype in response to mechanical stimuli.^[^ ^9^, ^21^ ^]^ In this study, an in‐house designed multiwell bioreactor was used to subject macrophages (THP‐1) to compressive load and shear. Conditioned media, derived from macrophages, were then collected and added to MSC pellet cultures. The experimental outline is depicted in **Scheme** **1**.

**Figure 1:**
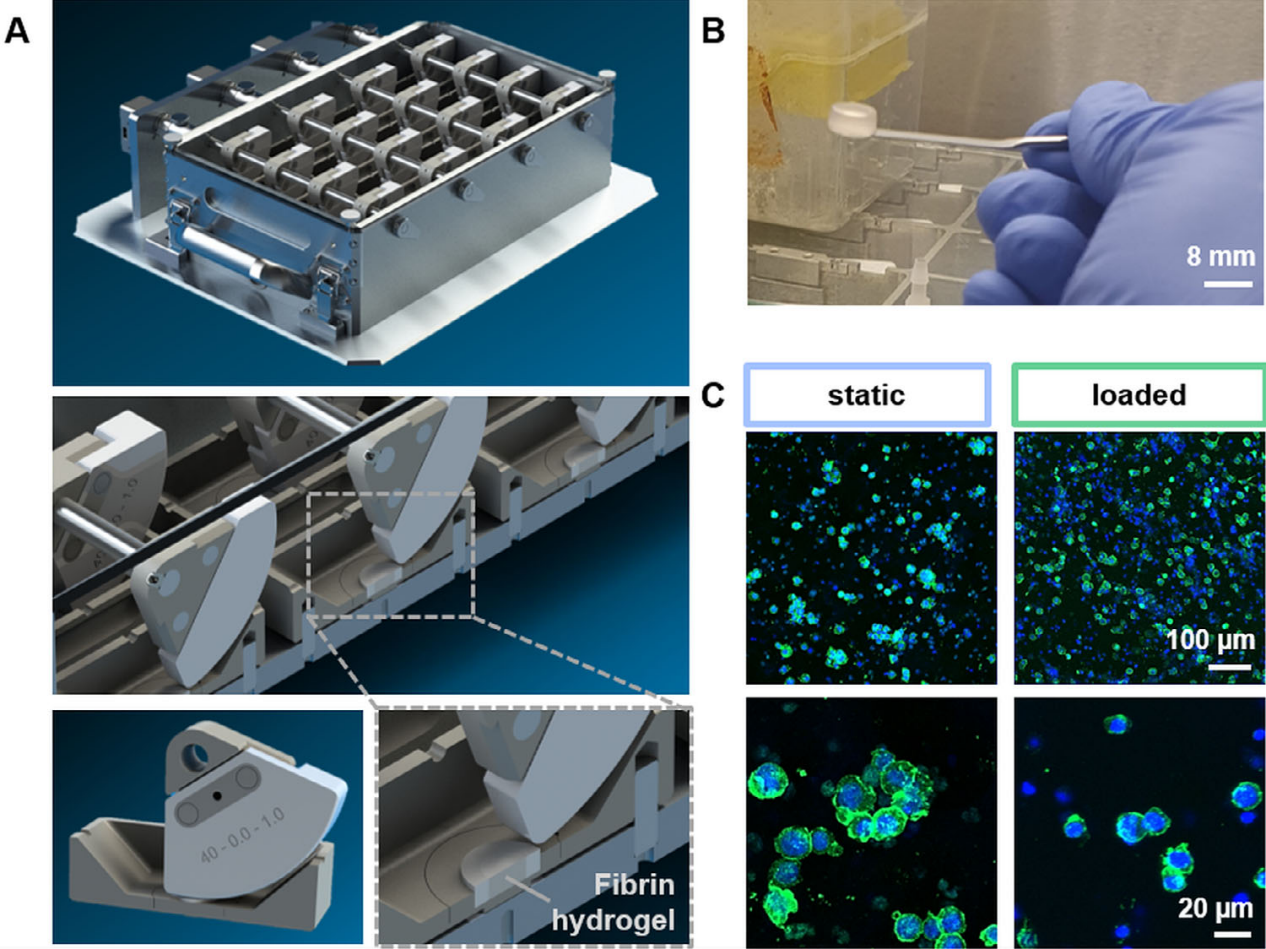
A) An in‐house designed multiwell bioreactor was used to culture THP‐1 macrophages in fibrin under compression and shear. The design allows for a simultaneous culture of 16 samples. A ceramic lever moves across the samples, thereby applying cyclic compression (20%) at a frequency of 1 Hz. B) Stable fibrin hydrogels were formed that were then placed into the sample holders displayed in A. C) Confocal microscopy images of THP‐1 macrophages cultured in fibrin hydrogels under static or mechanically loaded conditions. Actin cytoskeleton is displayed in green, and nuclei in blue.

Initially designed for chondrogenic differentiation of MSCs,^[^ ^29^ ^]^ the 16‐well multiaxial bioreactor was shown to be suitable to culture THP‐1 cells under 20% cyclic compression load at 1 Hz shear (**Figure** **1**A) for 1 h a day over 3 days. This loading regime was chosen with clinical application in mind, as the 1 Hz frequency represents a gentle walking pace that can be implemented during physiotherapy. Furthermore, previous studies in our group indicated an increased expression of *COL2A1, AGG, Sp7, TGFβ1, or TGFβ3* in MSC at 1 Hz and 20% compression, compared to shear and compression at 0.1 Hz, and 5% or 10%, respectively.^[^ ^30^ ^]^ Indicative for improved chondrogenesis and thus potentially endochondral ossification during bone fracture healing, the same parameters were applied in this study. Illustrated in Figure 1B, stable fibrin‐cell hydrogels were obtained at the chosen concentration and could be readily placed into the sample holders. Fibrin hydrogels were placed into sponge‐like poly(urethane) rings to provide structural support within the PEEK sample holder and allow for semi‐confined compression. Confocal microscopy images (Figure 1C) indicate comparable THP‐1 distribution in static or mechanically loaded conditions (data on cell metabolic activity displayed in Figure S1, Supporting Information).

Cytokines from macrophages are of high importance during bone fracture healing. The pro‐inflammatory factor TNF‐α was reported to act in a complex, dose‐dependent manner with stromal cell recruitment, migration, and osteogenic differentiation at low concentrations, yet inhibition thereof at higher concentrations.^[^ ^31^, ^32^ ^]^ The other most prominent factors, IL‐1β and IL‐6, are generally associated with inflammatory diseases such as, e.g., osteoarthritis and were reported to inhibit osteoblast differentiation. Targeting IL‐1β or IL‐6 as a therapeutic measure is therefore suggested to help in bone repair, by reducing osteoclast activity and tissue degeneration.^[^ ^33^, ^34^ ^]^ Interestingly, interleukin 8 (IL‐8), which is also considered a pro‐inflammatory factor, was shown to have an important role during endochondral ossification and induced an increased expression of *Col2a1* and *Acan*, or *Sox9* and *Col2a1*, respectively, in vivo.^[^ ^35^, ^36^ ^]^With respect to anti‐inflammatory factors, elevated levels of TGFβ in serum and in the hematoma (callus) were observed during early phases of bone healing in clinical studies, with a decrease after 2 to 8 weeks, indicating its involvement in a time‐dependent manner during bone‐union.^[^ ^37^ ^]^ Similarly prominent during early phases, inhibition of VEGF in a mouse model resulted in impaired intramembranous or endochondral ossification, underpinning the importance of vascularization for successful fracture healing.^[^ ^38^ ^]^ IL‐10, on the other hand, has a twofold effect on bone formation with reported inhibition of osteoclast activity and thus reduced bone degradation, and increased osteogenesis and mineralization.^[^ ^39^ ^]^ How these factors change under mechanical stimulation, in particular compression and shear is, however, poorly understood. In turn, chemically induced M1‐like (using LPS alone or a combination of LPS and IFNγ) or M2‐like (IL‐4, IL‐13, or a combination of both factors) polarization of murine or human primary cells or cell lines on 2D tissue culture treated poly(styrene) (TCPS) substrates is a well‐known concept. Such established models to induce distinct polarization phenotypes enable research on exogenous chemical factors and physical stimuli that might influence the switch from one phenotype to the other. LPS‐induced pro‐inflammatory M1‐like macrophages are well‐studied cells to assess anti‐inflammatory agents. The cell line THP‐1 in particular constitutes an excellent in vitro model to study different research questions,^[^ ^40^, ^41^ ^]^ but it was reported that inducing an anti‐inflammatory phenotype, with pronounced secretion of IL‐10, is often challenging.^[^ ^42^ ^]^ In our hands and on 2D, THP‐1 behaved similarly to these studies and we could confirm previous findings. On the gene level, distinct pro‐inflammatory phenotypes were identified, with *IL1B, TNF, NOS2*, and *IL6* being increased in LPS‐stimulated THP‐1. Genes encoding for hallmarks of anti‐inflammatory macrophages, *CD163, CD206, IL10*, and *CCL18*, were less clearly distinguishable. Cytokine secretion of three main factors (IL‐1β, IL‐6, and IL‐10) was in line with these observations, with no detectable IL‐10 secretion (results presented in Supporting Information, Figure S2).

**Figure 2:**
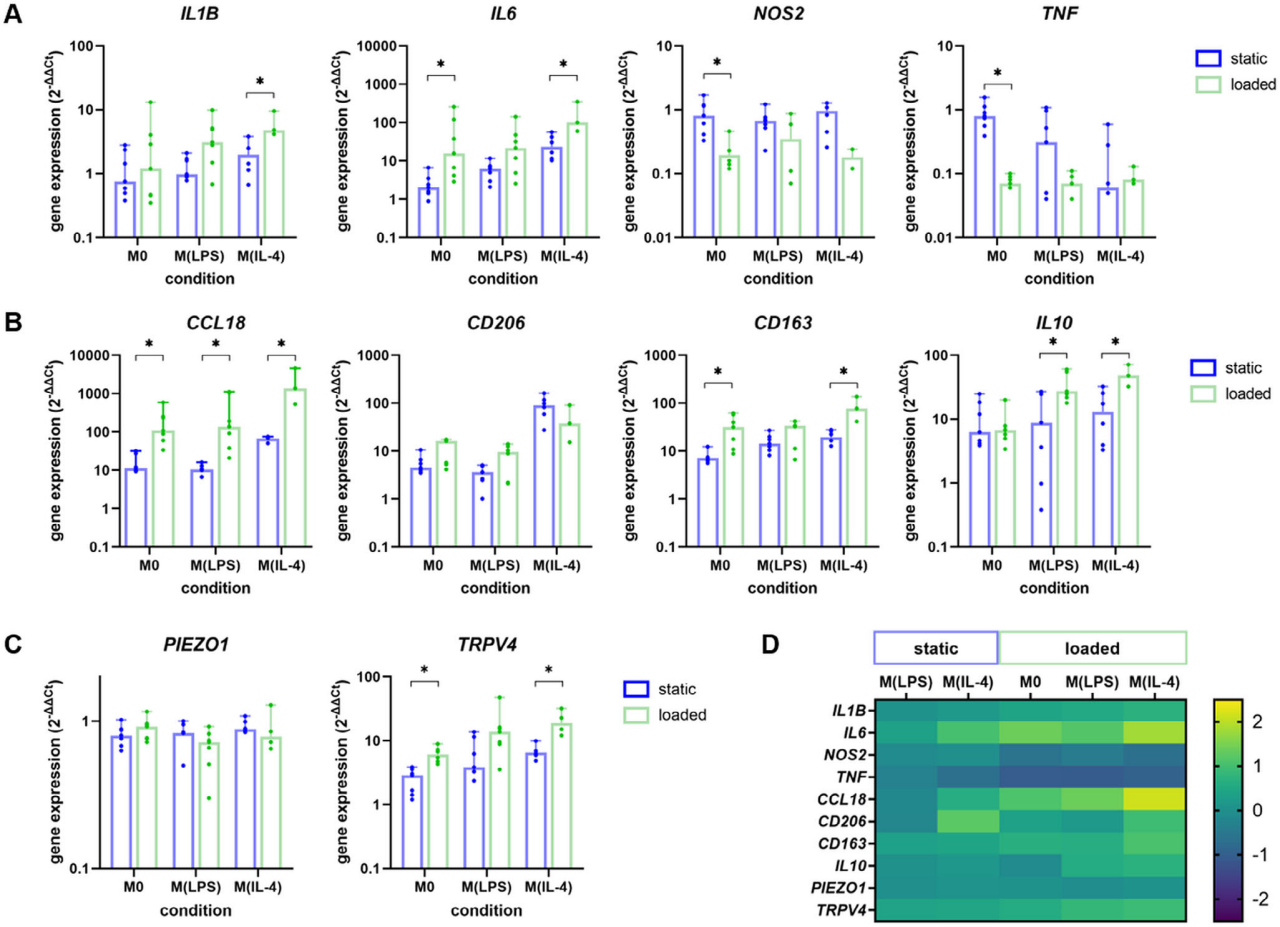
Gene expression analysis of macrophages cultured in fibrin hydrogels under static (blue) or mechanically loaded (green) conditions. A) Expression of genes encoding for pro‐inflammatory factors and B) anti‐inflammatory factors, C) mechanosensitive ion channels. Data are represented as median + 95% CI and normalized to the geometric mean of two housekeeping genes (ACTB and 18S) and to macrophages harvested on day 0 (2^−ΔΔCt^). Dots represent individual data points of two individual experiments. *p < 0.05, comparing static versus loaded culture for each group. D) Heat map summarizing gene expression levels relative to M0, cultured under static conditions. Data are presented on a logarithmic scale with base 10. The mechanical load shifted the macrophage phenotype toward an anti‐inflammatory phenotype with a more pronounced effect in IL‐4‐stimulated THP‐1 cells. This provides evidence for a synergistic effect of mechanical load and chemical stimulation toward an M2‐like phenotype.

In stark contrast, gene expression profiles of THP‐1 in fibrin hydrogels did not allow clear boundaries between one or the other phenotype when chemically stimulated (**Figure** **2**). It is likely that this more complex phenotype observed in 3D comes closer to the native one, with macrophages being found on a broad spectrum of different phenotypes and not necessarily in homogenous communities. Not only a 3D culture but fibrin or fibrinogen, in particular, have been reported to strongly influence macrophage phenotypes. Petrousek et al.,^[^ ^43^ ^]^ reported an immune‐regulatory effect of fibrin (at a concentration of 50 mg mL^−1^ fibrinogen and 2.5 U mL^−1^ thrombin), with a synergistic effect of fibrin and chemical stimulation (IL‐4 and IL‐13) on the expression of a M2a‐like phenotype with increased levels of *IL10* gene expression, and enhanced IL‐10 and VEGF secretion compared to THP‐1 on 2D. Hsieh et al.,^[^ ^44^ ^]^ cultured macrophages on fibrin hydrogels (bovine origin, 2 mg mL^−1^ fibrinogen and 0.4 U mL^−1^ thrombin), or on standard plastic culture dishes, and subjected both cultures to fibrinogen‐supplemented media. On fibrin, macrophages secreted high levels of IL‐10. Soluble fibrinogen, however, stimulated an increased secretion of TNF‐α. Remarkably, the effect of fibrin dominates the effect of fibrinogen in co‐culture systems, with macrophages being resistant to LPS and IFNγ stimulation, resulting in reduced TNF‐α secretion. In a study by Smiley, fibrinogen was also shown to increase the expression of *CCL2* which is often reported to be a marker for a M1‐like phenotype.^[^ ^45^ ^]^ Pointing in the opposite direction of an anti‐inflammatory effect of fibrin, Lackington et al.,^[^ ^46^ ^]^ incubated titanium‐based implants with blood and demonstrated increased levels of TNF‐α, IL‐6, and IL‐8 secreted by THP‐1 macrophages cultured on blood‐incubated samples with a fibrin‐rich network compared to pristine ones. Taken together, the effect of fibrin or fibrinogen on macrophage polarization is still elusive and most likely highly depends on the culture conditions, time of evaluation, and degree of fibrinogen‐thrombin crosslinking. Our results provide evidence for a fibrin‐dominated phenotype commitment compared to a chemically induced one.

Mechanical load, however, increased the expression of *ILB, IL6, CCL18, CD163*, or *IL10* compared to static culture in most conditions, while *NOS2* or *TNF* was downregulated under load. Platforms to culture macrophages under continuous flow, vibrational stimuli at different amplitude and frequency on 2D culture substrates, or tension on elastic membranes, are more common than systems to apply compression or shear, which limits the comparability of our results.^[^ ^9^, ^20^, ^21^ ^]^ On a Flexcell‐5000T Tension System, Tu et al., reported an increased expression of pro‐inflammatory factors in Raw264.6 cells at 15% stretch compared to lower percentage or static cultures.^[^ ^47^ ^]^ Wu et al. worked with different micro‐vibration regimes and identified a set of parameters (frequency 10 Hz, magnitude 0.45 g, and time 60 min) to suppress pro‐inflammatory factors IL‐1β or TNF‐α, with increased expression of IL‐10.^[^ ^48^ ^]^ Son et al., cultured macrophages under unidirectional laminar flow and reported on increased gene expression of pro‐inflammatory factors including *CCL2, IL1B*, and *TNF*.^[^ ^49^ ^]^ Anti‐inflammatory markers were, however, not investigated and it remains elusive whether a general upregulation under flow took place compared to static culture or whether it was specific to pro‐inflammatory factors. Without consensus on a distinct phenotype, or applied mechanical stimuli, there is general agreement, that mechanical stimuli have an effect on macrophage polarization. This has clinical relevance as the load across a fracture gap is poorly defined. Mechanical stimulation has been reported to act on many sensory units of different cells, comprising, but not limited to, integrins, the cytoskeleton, and mechanosensitive ion channels.^[^ ^47^, ^50^, ^51^ ^]^ Among others, *PIEZO1* and *TRPV4* are the most important genes encoding for mechanosensory ion channels in musculoskeletal tissues by maintaining bone homeostasis, controlling cartilaginous matrix deposition, osteoblast or chondrocyte proliferation and differentiation.^[^ ^52^, ^53^ ^]^ Further, multiple studies point toward *PIEZO1* or *TRPV4* as key players in mechanoregulated macrophage polarisation with increased susceptibility of macrophages to LPS and IFNγ with highly expressed *PIEZO1*.^[^ ^51^, ^54^, ^55^ ^]^ If *PIEZO1* was knocked out or downregulated, an M0 to M2 switch was reported. Conversely, activation of *TRPV4* in LPS‐stimulated macrophages resulted in the downregulation of pro‐inflammatory factors and increased IL‐10 secretion. Taken together, this suggests that triggering *PIEZO1* is pro‐inflammatory, while *TRPV4* expression leads to a more anti‐inflammatory phenotype. Here, *PIEZO1* was not affected by the chosen loading regime, while *TRPV4* was significantly upregulated in macrophages that were subjected to shear and compression in the M0 or the M(IL‐4) group, with a similar trend seen in the M(LPS) group. Based on previous literature, this would suggest the chosen loading pattern would overall reduce inflammation.^[^ ^56^ ^]^

To understand the underlying mechanisms within the here reported system, future studies should investigate the integrin‐cytoskeleton *YAP/TAZ* and RhoA/Rock pathways as well as the PIEZO1/NF‐κB/NLRP3 signaling cascade. Summarized in the heat map in Figure 2D, gene expression values are normalized to M0 static. Changes are more pronounced in IL‐4 stimulated conditions, indicative of a synergistic role of chemical stimulation and mechanical load on the expression of anti‐inflammatory factors.

**Figure 3:**
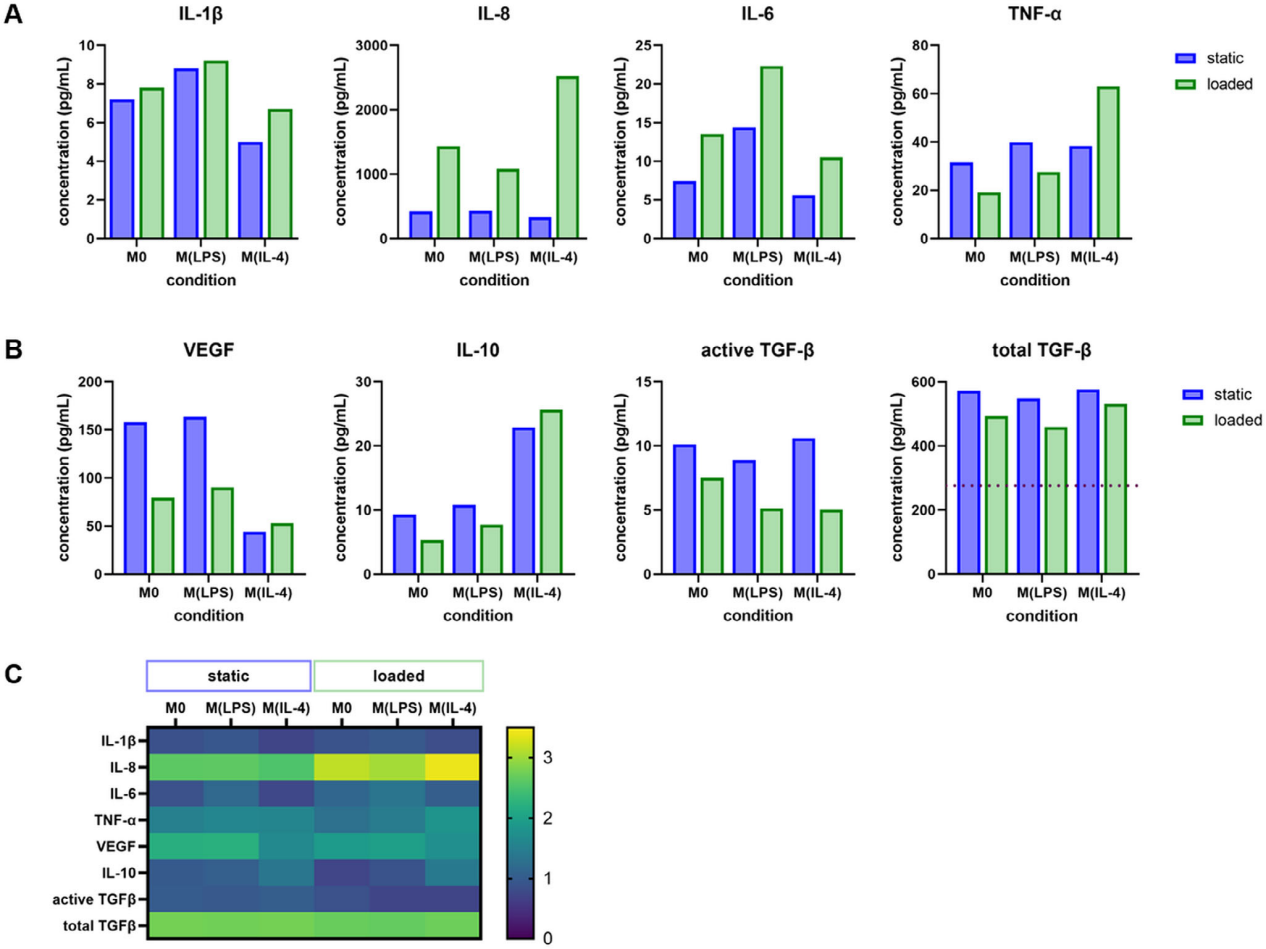
Cytokine secretion was assessed with ELISA. A) Pro‐inflammatory cytokines and B) anti‐inflammatory cytokines. ELISA analyses were conducted on media pooled from 2 independent experiments. FBS‐supplemented media has a considerably high concentration of latent TGFβ, which is detected with ELISA after acid activation. The background value is therefore displayed with a dashed line in the graph. Values in cell‐free media for all other cytokines were below the detection limit. C) Heat map summarizing cytokine secretion. Data are presented on a logarithmic scale with base 10. Mechanical load mainly increased the secretion of IL‐8 and IL‐6, and induced reduced secretion of VEGF and TGFβ.

Cytokine secretion of major factors influencing fracture healing was analyzed with Enzyme‐Linked Immunosorbent Assay (ELISA) and is reported in **Figure** **3**. Values of pooled conditioned media of two independent repeats with 3 to 4 scaffolds in each experiment are shown. Media was analyzed in the pooled form in order to characterize the components supplied to MSC further down the experimental line. Cytokine concentrations were generally lower in the loaded groups than in the static control with some exceptions: An increased IL‐8 secretion in all loaded groups, an increased TNF‐α secretion in M(IL‐4), and an increased IL‐6 expression in M(LPS) was observed. While the small sample number does not allow for statistical evaluation, differences among groups in IL‐6, IL‐8, IL‐10, and VEGF secretion are between 2‐ to 6‐fold, which is a biologically significant concentration difference. TGFβ is secreted in a latent, inactive form by cells, but can be activated under mechanical load by means of bioreactors.^[^ ^23^ ^]^ Different from previous studies with polyurethane scaffolds, mechanical load did not activate latent TGFβ, and a reduced concentration of active TGFβ, at the limit of detection, was measured in media derived from macrophages cultured under mechanical load. Total TGFβ (acid‐activated TGFβ prior to ELISA analysis) is also reduced under load, indicating that neither latent TGFβ activation, nor total TGFβ secretion was increased under mechanical load. Fetal bovine serum (FBS)‐supplemented media naturally contains high concentrations of TGFβ (≈300 pg mL^−1^), leading to values only marginally above background values in media derived from macrophages.

Heat maps in Figures 2 and 3 summarise the data on the gene or protein expression level, respectively, and provide evidence for a more complex phenotype in 3D or under mechanical load compared to standard 2D cultures with chemically induced polarization. Summarised in an excellent table by Ganguly and colleagues^[^ ^21^ ^]^ surface markers or cytokine secretion profiles follow distinct combinations, with M1, M2a, M2b, M2c, or M2d being reported as previously identified subtypes of macrophages. M2b is likely the most ambiguous type with pro‐ and anti‐inflammatory cytokine secretion (IL‐1β, IL‐6, TNF‐α, IL‐10) and increased expression of *IL10* and *CD163*. Based on this classification, our data suggest that macrophages under mechanical load undergo a transition to an M2b phenotype. TNF‐α, generally handled as a key factor in inflammatory diseases, is indeed secreted by M1 and M2b macrophages but downregulated under mechanical load in our experiments. In previous studies by Fahy and colleagues,^[^ ^27^ ^]^ primary monocytes or THP‐1‐Blue reporter cells were cultured in agarose hydrogels under a comparable loading regime (1 h stimulation per day over 3 days, at 1 Hz and 10% dynamic compression), leading to pro‐inflammatory genes and cytokines being upregulated in M0‐like macrophages as well as M(IL‐4) macrophages compared to the static control. This direct comparison indicates the importance of loading parameters and biomaterial compositions, which has not been addressed in systematic studies to date. Further research is needed to investigate the different phenotypes at different time points, in distinct hydrogels, and under specific loading regimes to gain a better understanding of the effect of mechanical load on macrophage polarisation. Specifically, parameters defining optimal load or overload need to be identified and validated in future studies involving key players during bone healing, including, but not limited to chondrocytes, osteoblasts, or macrophages. Our main aim, however, was to elucidate the effect of conditioned media from different macrophage cultures on MSC differentiation to assess whether initial immunomodulatory events influence endochondral ossification.

### 2.2 Conditioned Media from Mechanically Stimulated Macrophages Triggers Early Events in Endochondral Ossification

In the second part of our model system, we cultured MSC pellets in conditioned media derived from macrophages. These macrophages were cultured either under static or dynamic conditions and were supplemented with LPS and IFNγ, or IL‐4. Different from reports on 2D models under less complex conditions, gene expression of macrophages cultured under distinct conditions and their secretomes did not follow a clear classification into pro‐ or anti‐inflammatory phenotypes (Figures 2 and 3). To account for this, we also conducted a small series of complementary experiments with MSC pellets cultured in conditioned media from macrophages cultured on 2D with a more distinct gene expression profile (Supporting Information, Figures S2–S4).

**Figure 4:**
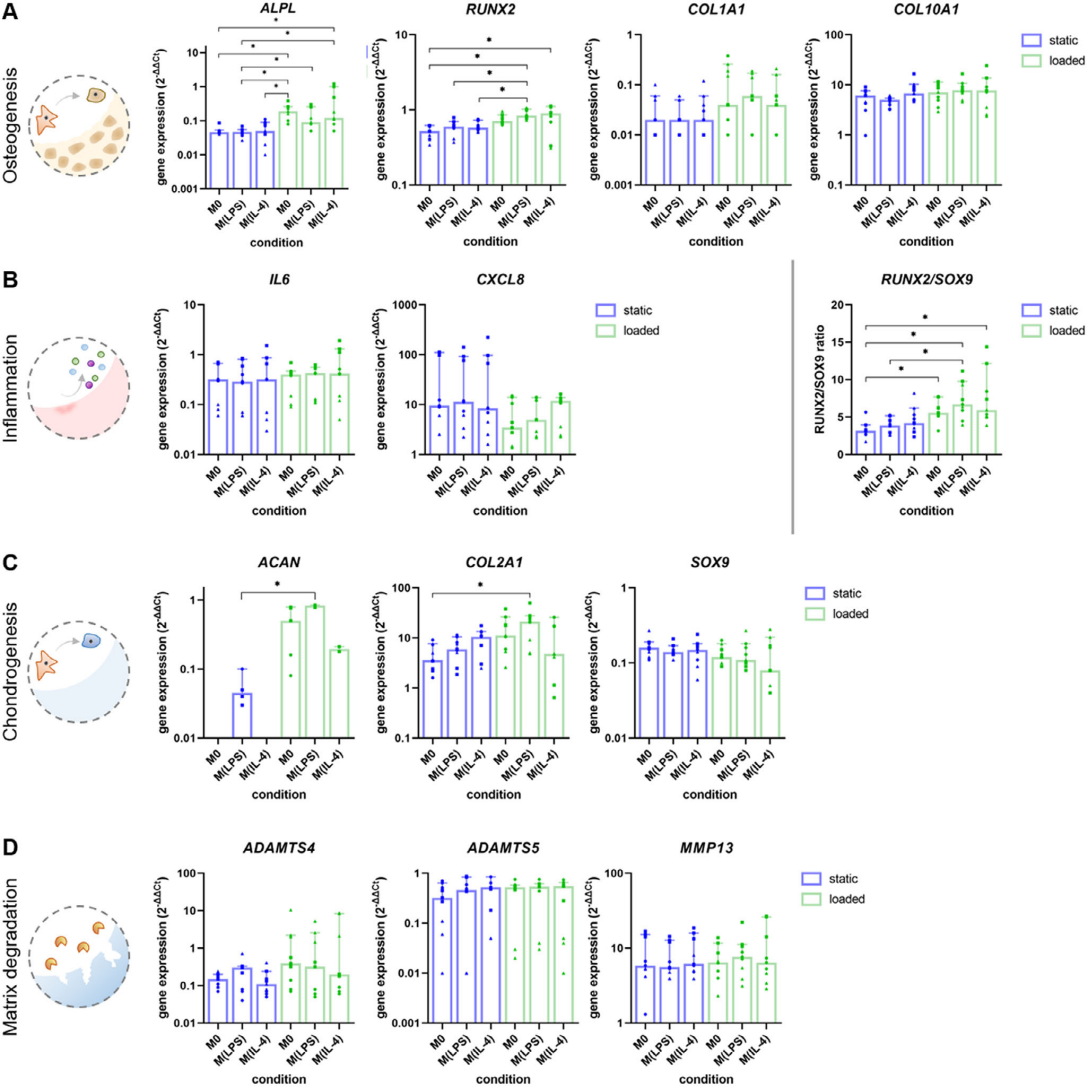
Gene expression profile of MSC pellets cultured with conditioned media derived from macrophages cultured under static (blue) or mechanically loaded (green) conditions. M0, M(LPS), or M(IL‐4) indicate the chemical stimulation. A) genes encoding for osteogenic markers, B) inflammatory proteins, C) chondrogenic markers, and D) matrix‐degrading enzymes. Results are presented as median + 95% CI (bars and error bars). Individual data points are plotted as dots (donor 1), rectangles (donor 2), or triangles (donor 3). Gene expression was normalized to the housekeeping gene RPLP0 and MSC pellets harvested after 24 h (2^−ΔΔCt^), *p < 0.5.

**Figure 5:**
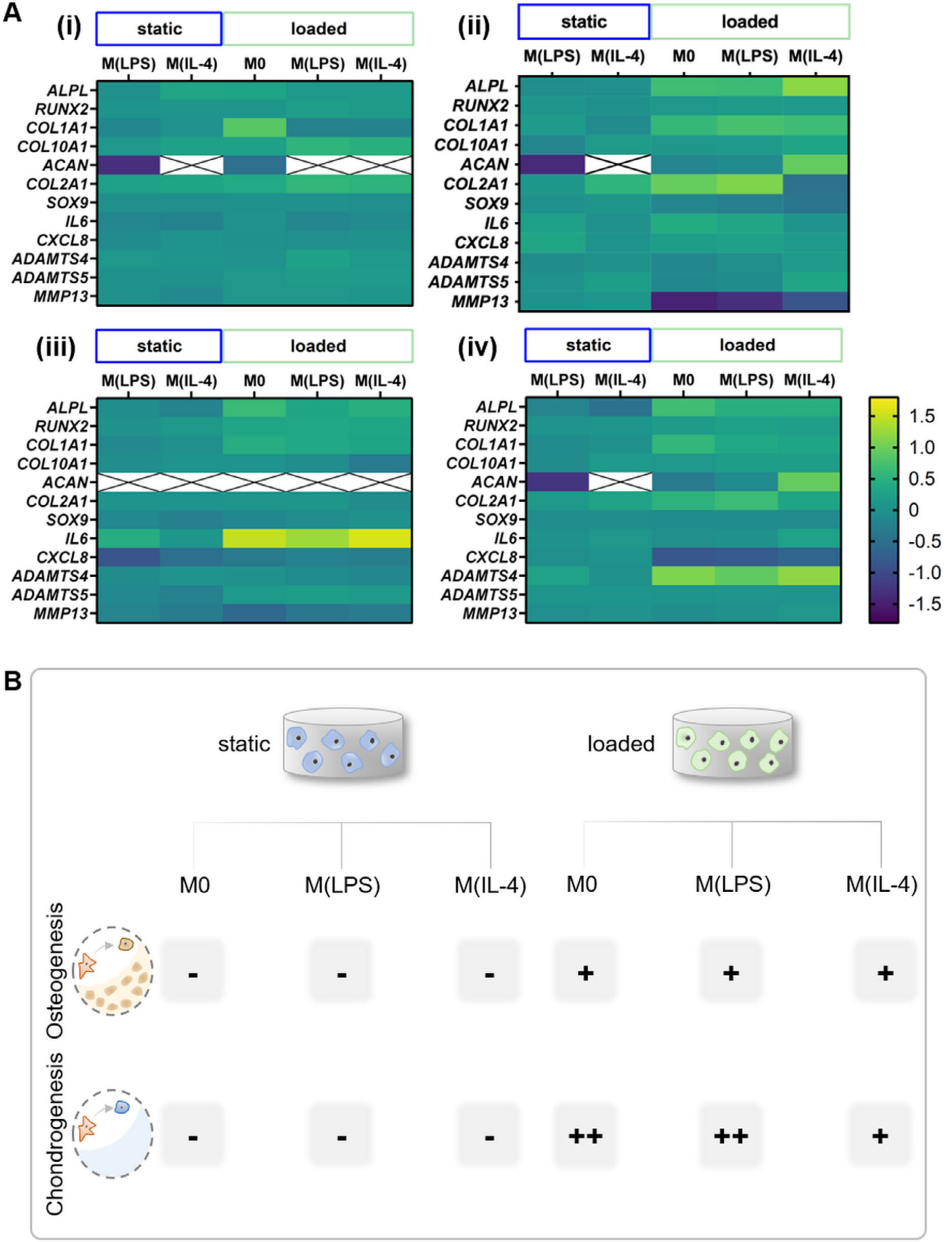
A) Heat maps of gene expression profiles for i) donor 1, ii) donor 2, iii) donor 3, and iv) mean values of all donors. Values are represented relative to M0‐static and plotted on a logarithmic scale with base 10. No ACAN was expressed in MSC cultured with a conditioned medium from M0 macrophages cultured under static conditions. Values for this gene are therefore absolute and not relative to M0. B) Schematic summary of the main findings. Conditioned media derived from macrophages cultured under mechanical load increased the expression of chondrogenic and osteogenic genes in MSC pellets after 9 days.

By standard RT‐qPCR, a set of 12 genes, encoding for osteogenic hallmarks (*ALPL, RUNX2, COL1A1*, and *COL10A1*), chondrogenic hallmarks (*ACAN, COL2A* *1*, and *SOX9*), matrix‐degrading enzymes (*ADAMTS4, ADAMTS5*, and *MMP13*), or inflammatory markers (*IL6* and *CXCL8*) were assessed (**Figure** **4**, additional groups displayed in Supporting Information Figure S4). While markers cannot be strictly associated with one or the other mechanism, the chosen genes play an important role during in vitro differentiation of MSC (osteogenic markers and chondrogenic markers) or are associated with an inflammatory environment, for example, osteoarthritis or fracture healing. As reported in Figure 4A, macrophage secretomes had a significant effect on the expression of early osteogenic markers *ALPL* and *RUNX2*. MSC treated with conditioned media from mechanically stimulated macrophages expressed significantly increased amounts of *ALPL*, while no differences were observed among MSC treated with conditioned media derived from M0, M(LPS), or M(IL‐4) cultured under static conditions. In line, *RUNX2* gene expression was increased in pellets treated with media from mechanically stimulated cells compared to static groups. *RUNX2/SOX9* ratio, an early marker for osteogenic differentiation^[^ ^57^, ^58^ ^]^ was also increased for MSC treated with conditioned media derived from mechanically loaded macrophages. Interestingly, there were no significant differences in whether macrophages were chemically treated or not. Later markers, genes encoding for extracellular matrix proteins collagen type 1 and collagen type 10, were not upregulated in any condition compared to M0. Generally expressed at a later stage, changes might have been observed at day 14 or 21, but not during the early time points that were the focus of this study. Genes encoding for chondrogenic differentiation and cartilage matrix (*ACAN* and *COL2A1*) were significantly increased in pellets treated with conditioned media derived from M(LPS) macrophages cultured under mechanical load compared to the static condition. For inflammatory factors or matrix‐degrading enzymes, our data did not reveal any significant differences. As is common for work with primary human cells derived from different donors, large variations among donors were observed. For genes where differences among donors were small, significant differences were observed which was evidently not the case for genes with large differences among donors. To account for donor variations, relative values for each donor relative to M0 are presented in heatmaps in **Figure** **5**. Increasing sample size or pre‐screening donors at times helps solve this issue, but the data obtained would be less clinically relevant.

Macrophage secretomes have been previously shown to have an effect in a variety of experimental settings with different clinical implications. Lentilhas‐Graça and team reported on distinct effects on neuronal growth and survival, with alternatively activated macrophages promoting axonal regeneration.^[^ ^59^ ^]^ In a co‐culture model with THP‐1 and MSC, Kolliopoulos^[^ ^41^ ^]^ underpinned the fact that THP‐1 stimulated the osteogenic potential of MSCs in a paracrine manner, while MSC also had an immunomodulatory effect on THP‐1. Generally speaking, the crosstalk between THP‐1 and MSC has long been appreciated with many in vitro and in vivo models elucidating distinct aspects.^[^ ^60^ ^]^ Zhou et al.,^[^ ^61^ ^]^ designed an IL‐4 functionalized hydrogel to induce an M2‐like macrophage phenotype. Conditioned media from this culture was shown to increase osteogenic differentiation, hypothetically by increased bone morphogenetic protein (BMP) secretion. Work by Yong and colleagues used exosomes derived from macrophages cultured on different materials ^[^ ^62^ ^]^ and identified different micro RNAs (miRNAs) that might be responsible for the observed effects. A polarization toward an M2 phenotype (in response to material properties) was demonstrated to accelerate bone formation further down the line. Zhang^[^ ^63^ ^]^ comparably provided evidence that macrophages triggered an M2 phenotype (by use of IL‐4) promoted osteogenesis, while macrophages treated with LPS and IFNγ inhibited osteogenesis. In co‐culture models of macrophages and MSC, macrophages induced to an M2 phenotype were also shown to increase osteogenic differentiation of MSC.^[^ ^64^, ^65^ ^]^ The underlying cause for the observed changes in these publications was mainly attributed to cytokines also assessed in our study (IL‐1β, IL6, IL‐8, TNF‐α, TGFβ, VEGF, or IL‐10). Distinct levels of circulating TGFβ at specific time points for example were reported to be a marker for delayed bone healing or nonunion, while it also is an important growth factor to stimulate chondrogenesis. Further yet, it plays a dual role in both bone resorption (by acting on osteoclasts) and bone‐forming events.^[^ ^37^, ^66^ ^]^ In vitro, chondrogenesis is often induced with 5 to 10 ng mL^−1^ TGFβ. Here, the measured values in conditioned media are significantly lower and therefore unlikely responsible for increased chondrogenesis. (Displayed in Figure S5 (Supporting Information) are control conditions, e.g. chondropermissive medium (CpM) or chondrogenic medium (CgM), each mixed with complete RPMI for comparison). Aside from these considerations, TGFβ secretion decreased after mechanical stimulation, while expression of *ACAN* and *COL2A1* were increased in M(LPS)‐loaded condition, which can therefore hardly be attributed to an increased level of this factor. VEGF has not only been shown to accelerate bone growth by vascularization but also directly acts on osteoblasts and stimulates both endochondral ossification and intramembranous ossification. Impaired vascularization has been associated with impaired bone growth and timing, as well as the concentration of VEGF was crucial for successful bone healing.^[^ ^67^, ^68^ ^]^ The importance of VEGF has furthermore been underpinned by VEGF inhibition in a mouse model, with improved osteogenesis when VEGF was exogenously supplied.^[^ ^38^ ^]^ In our hands, macrophages subjected to load secreted less VEGF than the static control, and M(IL‐4) macrophages secreted even less. The main anti‐inflammatory factor, IL‐10, which is also increasingly secreted in M(IL‐4)‐loaded conditions in our work, has been known to promote the expression of *OPN* and inhibit *RANKL*, which thereby reduces osteoclast activity, and enhanced osteogenesis and mineralization.^[^ ^39^, ^69^ ^]^ Here, the secretion of these anti‐inflammatory markers, IL‐10 and VEGF, correlated negatively with increased gene expression of *ALPL, RUNX2, ACAN*, or *COL2A1* in that these four genes were increased in MSCs treated with conditioned media derived from mechanically stimulated macrophages.

With respect to pro‐inflammatory factors, in mouse KO models, delayed endochondral maturation was shown when the TNF‐α receptor was not present, or in IL‐6 KO mice. Additionally, TNF‐α and IL‐6 were shown to recruit osteoblasts to the site of injury.^[^ ^31^ ^]^ A review article by Karnes et al.^[^ ^32^ ^]^ emphasized that TNF‐α, along with progenitor cells, is the main determinant of successful bone union, indicated by reduced TNF‐α levels in diseases that are associated with impaired bone healing. TNF‐α peaks between day 1 to 3 after fracture followed by a reduction and another peak at a later phase during the transition of chondrogenesis to osteogenesis. Different from the above effects, blocking IL‐6 has also been suggested as a method to prevent bone degeneration and improve bone repair,^[^ ^33^ ^]^ for example in osteoarthritis where an inflammatory environment persists. As reported in these in vitro studies, exogenously supplemented IL‐6 led to reduced gene expression of *RUNX2, OSX, OCN*, or *ALP* activity. In summary, concentration and time‐dependent effects are likely to play a major role in the observed differences. Our results indicate an increased TNF‐α production in M(IL‐4) and increased *ALPL* and *RUNX2* expression in MSC pellets treated with conditioned media from loaded macrophages. The factor most affected and increased under mechanical load was IL‐8. IL‐8 has been associated with natural regenerative processes and was shown to increase *ACAN* and *COL2A1* expression, stimulate the expression of BMP receptors, and thereby improve the susceptibility of progenitors to BMP‐2.^[^ ^35^ ^]^ In our study, the expression of IL‐8 in M(LPS) under mechanical stimulation positively correlated with an increased gene expression of *ACAN* and *COL2A1* in MSC pellets. These 12 assessed genes provide the first indications of the chondrogenic or osteogenic differentiation of MSCs cultured with conditioned media derived from macrophages. To gain more in‐depth knowledge of underlying processes and broaden the investigated gene or protein expression profile, future studies should focus on RNA sequencing and proteomics. Thereby, the limitations of this study could be overcome and further information will be gained.

### 2.3 Conditioned Media had no Significant Effect on Sulfated Glycosaminoglycan per DNA Production

**Figure 6:**
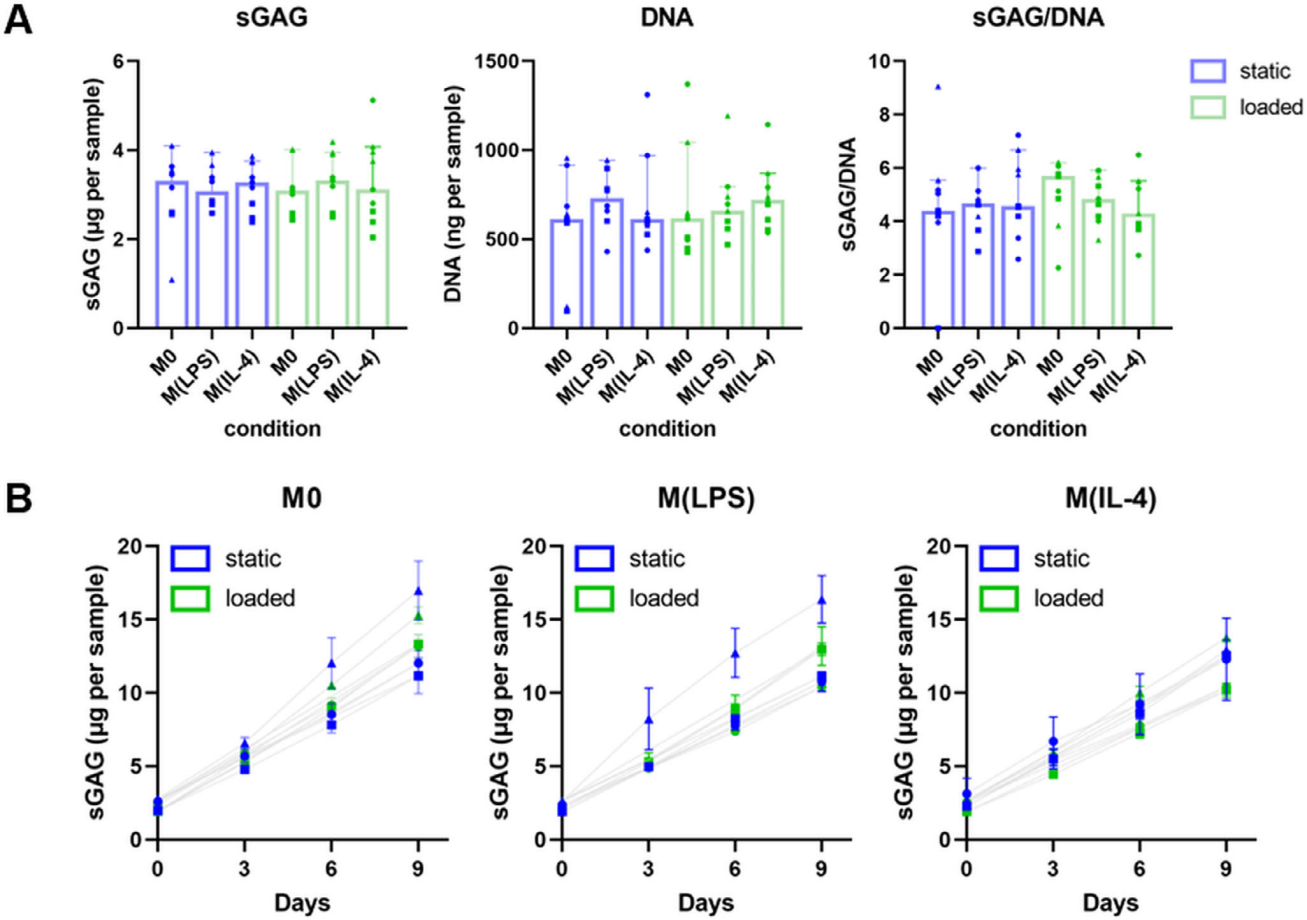
Extracellular matrix formation in MSC pellet cultures. A) Quantification of DNA, sGAG, sGAG/DNA in pellets after 9 days. Results are reported as median + 95% CI (bars and error bars). Individual data points are plotted as dots (donor 1), rectangles (donor 2), or triangles (donor 3). B) quantification of sGAG secreted to the media. Results are plotted as a median ± 95% CI of three pellets per donor per day with dots (donor 1), rectangles (donor 2), or triangles (donor 3).

With a focus on endochondral ossification and the early time point of 9 days, matrix deposition as an additional chondrogenic hallmark was characterized. Specifically, we characterized the deposition of sulfated glycosaminoglycan (sGAG). sGAG is one of the major components of the cartilage extracellular matrix and can be assessed in a spectrometric assay after pellet digestion. sGAG production correlates with a chondrogenic phenotype as well as cell number, to which end sGAG was normalized to DNA. sGAG, DNA, sGAG/DNA, and sGAG in the medium are displayed in **Figure** **6**. No significant differences among the conditions were observed. sGAG usually correlates with gene expression of *ACAN* and *COL2A2* and was therefore expected to be increased in pellets treated with conditioned medium from M(LPS)‐loaded. Pronounced sGAG deposition is often reported after a prolonged culture period and in TGFβ‐supplemented culture medium (additional experimental groups displayed in Supporting information Figure S4).

## 3 Conclusion

In conclusion, we developed a bi‐phasic in vitro model to a) culture macrophages under compression and shear and b) assess the uni‐directional cross‐talk between macrophages and MSC. Gene expression profiles encoding for osteogenic or chondrogenic hallmarks, as well as matrix‐degrading enzymes and inflammatory factors, were assessed after 9 days in culture, to simulate early events in bone fracture healing. Our results provide evidence for the immunomodulatory effects of i) a 3D environment in a fibrin hydrogel and ii) compression and shear. This combinatorial effect was manifested both on the gene expression level and protein/cytokine secretion. Early events during MSC differentiation were shown to be influenced by macrophage secretomes with increased expression of *ALPL, RUNX2, COL2A1*, and *ACAN* in cultures subjected to secretomes from mechanically stimulated macrophages. Previous studies highlighted that conditioned media, extracellular vesicles, or nanovesicles from macrophages of different polarization were shown to have an effect on osteogenesis. In the majority of studies, a general trend toward increased osteogenesis in response to M2‐polarized macrophages was observed. Importantly, however, hallmarks for osteogenesis were often assessed at later time points compared to our 9‐day period (14 or 21 days). Gene expression levels and cytokine secretion in our model point toward a more complex macrophage phenotype than traditionally reported and it cannot be concluded whether the observed effects in MSC are due to an M1 or an M2 phenotype. Nevertheless, osteogenic hallmarks as well as markers for chondrogenesis are increased on the gene level in MSC pellets that were treated with conditioned media from macrophages cultured under compression and shear. The combination of different factors, especially at critical concentrations, is what ultimately decides between a successful onset of differentiation or not. Interconnected signaling pathways, factors acting in concert in synergistic or antagonistic ways, and technical limitations with in vitro as well as in vivo models render it difficult to identify precise functions and effects of one or the other factor. With this bi‐phasic model, we contributed to a better understanding of the effect of mechanical load on macrophage polarization and their involvement during bone fracture healing.

## 4 Experimental Section

### Chemicals and Cell Culture Lab Ware

If not stated differently, chemicals were purchased from Sigma‐Aldrich/Merck (Buchs, Switzerland) and used as received without any further purification. Cell culture flasks and well plates were purchased from Techno Plastic Products (TPP, Trasadingen, Switzerland), RNAse and DNAse free. Cell culture media and supplements were purchased from Gibco, Invitrogen, and ThermoFisher Scientific, USA. All media were prepared from powder, supplemented with the respective additives and sterile filtered through a cellulose membrane.

### THP‐1 Expansion and Culture Under Mechanical Load

THP‐1 cells (human monocytes, ECACC, Sigma) were expanded in suspension in RPMI 1640 medium with HEPES (Gibco 13018–031), supplemented with 0.85 g L^−1^ Sodium Bicarbonate (NaHCO_3_), 10% v/v fetal bovine serum (FBS, 35‐079‐CV, Corning), 100 U mL^−1^ Penicillin and 10 µg mL^−1^ Streptomycin (Pen/Strep, 15140‐122, Gibco) (referred to as complete RPMI). Upon defrosting, THP‐1 was recovered in RPMI 1640 supplemented with 20% FBS and Pen/Strep. Culture density was maintained below 1·10^6^ cells mL^−1^ with regular medium addition 2 to 3 times per week.

Fibrinogen (F4883‐5G, 96% clottable fraction) was reconstituted in 0.9% w/v NaCl in milliQ water at 50 mg mL^−1^. Thrombin (T4393‐100UN) was reconstituted in 0.1% w/v Bovine Serum Albumin (BSA) to a final concentration of 100 IU mL^−1^. Fibrin gels were prepared in complete RPMI medium supplemented with 5 µM ε‐aminocaproic acid in PBS (EACA, A7824‐25G) at final concentrations of 25 mg mL^−1^ fibrinogen, 2 IU mL^−1^ thrombin and 10·10^6^ THP‐1 mL^−1^ (2·10^6^ cells per scaffold measuring 4 mm in height and 8 mm in diameter). All components were kept on ice to prolong the time to gelation. Cell‐fibrin gels were cast into sterile silicone molds, let to gel at 37 °C in a humidified incubator for 30 min, and transferred to 24‐well plates for subsequent culture.

Monocyte to macrophage transition within hydrogels was induced by 60 ng mL^−1^ phorbol‐12‐myristate‐13‐acetate (PMA, P8139) for 24 h, followed by a 72‐h rest period in complete RPMI. A pro‐inflammatory phenotype was induced by adding 100 ng mL^−1^ Lipopolysaccharide from *Escherichia coli* O55:B5 (LPS, L6529), and 10 ng mL^−1^ human recombinant interferon‐gamma (IFN‐γ, 300‐02‐11UG, PeproTech, ThermoFisher Scientific, USA) to RPMI complete; referred to as M(LPS). An anti‐inflammatory phenotype was induced by adding 20 ng mL^−1^ recombinant human interleukin 4 (IL‐4, 200‐04‐100U, PeproTech, ThermoFisher Scientific, USA) to RPMI complete, referred to as M(IL‐4). Polarization was induced for 48 h. Subsequently, samples were distributed into two groups, static control, and mechanical load. For the latter, cell‐fibrin hydrogels were subjected to 10% compressive pre‐load, followed by 20% cyclic compression at 1 Hz shear rate for 1 h a day over 3 days in an in‐house built multi‐well bioreactor. The multiwell bioreactor design and function were previously reported by the group.^[^ ^29^ ^]^ In brief, fibrin‐cell hydrogels were placed in a Poly(ether ether ketone) (PEEK) based sample holder and kept in place with a flexible, porous poly(urethane) ring. Compression and shear were applied with an oscillating lever of polished ceramic. 16 individual samples can be subjected to identical mechanical loads, allowing for multiple samples to be cultured simultaneously. Here, *n* = 5 samples of each condition (M0, M(LPS), or M(IL‐4)) were cultured per experiment, with N = 2 individual experiments. Media was collected the next day and stored at −20 °C for further use. Three cell‐fibrin samples per condition were harvested and stored at −20 °C for subsequent qRT‐PCR analysis.

In an initial experiment, focusing on short‐term culture (3‐day culture) as well as excessive loading (5‐day culture with a total of 8 h mechanical stimulation) cell metabolic activity of THP‐1 in fibrin gels was assessed with Cell Titer Blue (Promega Corporation, Madison WI, USA), following the manufacturer's instructions. Absorbance was measured at λ = 570 nm on a TECAN Infinite 200 PRO M Plex microplate reader using the Magellan software (Tecan Group AG, Switzerland).

Subsequently, fibrin‐cell constructs were fixed in 4% v/v formaldehyde in PBS and stained for actin (Phalloidin Alexa 488‐conjugated, molecular probes, ThermoFisher Scientific, USA, 8.25 nM in PBS) and nuclei (4′,6‐Diamidin‐2‐phenylindol, DAPI, 2 µg mL^−1^ in PBS). Images were acquired on a laser scanning confocal microscope (LSM800, Carl Zeiss AG, Germany).

### Quantification of Secreted Cytokines with Enzyme‐Linked Immunosorbent Assays (ELISA)

To assess cytokine release from THP‐1 cells cultured under different conditions, media were collected at the end of the culture, static or loaded, and stored at −20 °C. Media from all samples per condition were pooled to supplement pellet cultures of different donors in subsequent experiments with the same formulation. Concentrations of interleukin 1‐beta (IL‐1β), interleukin 6 (IL‐6), interleukin 8 (IL‐8), interleukin 10 (IL‐10), transforming growth factor beta (TGFβ), tumor necrosis factor alpha (TNFα), or vascular endothelial growth factor (VEGF) in the supernatant were analyzed with ELISA (DuoSet ELISA, biotechne, R&D SYSTEMS, USA) following the manufacturer's instructions. TGFβ was analyzed with or without acid activation to quantify the available active form after mechanical loading, or the total TGFβ, respectively.

### Expansion and Chondrogenic Pellet Culture of Human Mesenchymal Stromal Cells

Mesenchymal stromal cells (MSC) were isolated from human bone marrow aspirates after informed consent and ethical approval (Ethik‐Kommission der Albert‐Ludwigs‐Universität Freiburg, EK‐326/08) using Ficoll (Histopaque‐1077) density gradient and plastic adhesion following previously published protocols from the group.^[^ ^70^ ^]^ Donors were male, 80 years (D#1), female 73 years (D#2), and female 80 years (D#3). MSC was expanded in Minimum Essential Medium alpha (α‐MEM, 12000–063, Gibco) supplemented with 2.2 g L^−1^ NaHCO_3_, 10% FBS and 5 ng mL^−1^ fibroblast growth factor‐basic (FGF‐b, 30R‐AF015, Fitzgerald Industries International, Biosynth), and 100 U mL^−1^ Penicillin and 10 µg mL^−1^ Streptomycin. MSC were split at 80% confluence and used in passages 3 or 4.

Chondropermissive medium (CpM) consisted of Dulbecco's Modified Eagle's Medium, high glucose (52100‐02, Gibco), 3.7 g L^−1^ NaHCO_3,_ 0.11 g L^−1^ sodium pyruvate, ITS+ Premix Universal Culture Supplement (ITS+, 354 352, Corning), 1x Non‐essential amino acids (NEAA, 11140‐035), 50 µg mL^−1^ L‐Ascorbic acid 2‐(dihydrogen phosphate) magnesium salt (AA‐2P, A8960), 100 nM Dexamethasone (Dex, D1756). Chondrogenic medium (CgM) consisted of the above, supplemented with 10 ng mL^−1^ transforming growth factor‐beta (TGFβ_1_, 30R‐AT072, Fitzgerald Industries International, Biosynth).

250 000 cells in 200 µL CpM were seeded in individual wells of V‐shaped 96‐well plates (Greiner‐CELLSTAR‐96‐well plates), and centrifuged at 1200 g for 5 min to form pellets. After 24 h, media was changed to the respective groups, followed by media change every 3 days. **Table** **1** provides information on the different media formulations. Conditioned medium from M0, M(LPS) or M2(IL‐4) macrophages, cultured under mechanical load or static, was mixed at a 1:2 ratio with CpM or CgM, respectively. As a control, complete RPMI mixed at a 1:2 ratio with CpM or CgM was used.

**Table 1:** List of different media compositions used to culture MSC pellets.

CpM Mixed With Media From	Culture Condition	Sample ID / Graphical Representation
M0	Static	M0, blue
	Loaded	M0, green
M(LPS)	Static	M(LPS), blue
	Loaded	M(LPS), green
M(IL‐4)	Static	M(IL‐4), blue
	Loaded	M(IL‐4), green
Control Groups		
CpM	n.a.	CpM
CpM and RPMI	n.a.	50% CpM
CgM	n.a.	CgM
CgM and RPMI	n.a.	50% CgM

### Quantitative Real‐Time Polymerase Chain Reaction (qRT‐PCR)

mRNA from THP‐1 cells or MSC pellets was isolated by use of an RNeasy mini kit (Qiagen, The Netherlands). Cells were lysed in 350 µL RLT‐lysis buffer in a TissueLyser2 (Qiagen, 30 Hz, 3 × 5 min) with 5 mm diameter metal balls (Martin & C, Perosa A, Italy) added to each sample. mRNA isolation was performed following the manufacturer's instructions. mRNA quantity and quality were assessed on a NanoDrop (ThermoFisher Scientific Inc., USA). Transcription to cDNA was performed with a High‐Capacity cDNA Reverse Transcription Kit (4368814, Applied Biosystems) in a thermocycler. qRT‐PCR was performed with TaqMan primer/probes or ready‐to‐use assays as indicated in **Table** **2** on a QuantStudio 7 Pro Real‐Time PCR System (ThermoFisher Scientific Inc., USA). Expression of specific genes in THP‐1 cells was normalized to the geometric mean of two housekeeping genes 18S and ACTB and reported as 2^−Δ∆Ct^ relative to samples harvested on day 0. Results are from *n* = 2 to 4 samples per condition from N = 2 individual experiments. Expression of specific genes in MSC was normalized to the housekeeping gene RPLP0 and reported as 2^−Δ∆Ct^ relative to pellets harvested after 24 h. Results are from *n* = 3 pellets per condition from 3 individual experiments (3 donors).

**Table 2:** List of assessed genes and corresponding primer/probe sequences or assay‐on‐demand identification codes.

Protein Encoded for, and Gene (Primer, Probe)		
Aggrecan (*ACAN*)	Forward	5′‐AGTCCTCAAGCCTCCTGTACTCA‐3′
	Reverse	5′‐CGGGAAGTGGCGGTAACA‐3′
	Probe	5′‐CCGGAATGGAAACGTGAATCAGAATCAACT‐3′
Collagen type 1 (*COL1A1*)	Forward	5′‐CCCTGGAAAGAATGGAGATGAT‐3′
	Reverse	5′‐ACTGAAACCTCTGTGTCCCTTCA‐3′
	Probe	5′‐CGGGCAATCCTCGAGCACCCT‐3′
Collagen type 2 (*COL2A1*)	Forward	5′‐GGCAATAGCAGGTTCACGTACA‐3′
	Reverse	5′‐GATAACAGTCTTGCCCCACTTACC‐3′
	Probe	5′‐CCTGAAGGATGGCTGCACGAAACATAC‐3′
Collagen type 10 (*COL10A1*)	Forward	5′‐ACGCTGAACGATACCAAATG‐3′
	Reverse	5′‐TGCTATACCTTTACTCTTTATGGTGTA‐3′
	Probe	5′‐ACTACCCAACACCAAGACACAGTTCTTCATTCC‐3′
Matrixmetalloproteinase 13 (*MMP13*)	Forward	5′‐CGG CCA CTC CTT AGG TCT TG‐3′
	Reverse	5′‐TTT TGC CGG TGT AGG TGT AGA TAG‐3′
	Probe	5′‐CTC CAA GGA CCC TGG AGC ACT CAT GT‐3′
60S acidic ribosomal protein P0 (*RPLP0*)	Forward	5′‐TGGGCAAGAACACCATGATG‐3′
	Reverse	5′‐CGGATATGAGGCAGCAGTTTC‐3′
	Probe	5′‐AGGGCACCTGGAAAACAACCCAGC‐3′
Runt‐related transcription factor 2 (*RUNX2*)	Forward	5′‐AGCAAGGTTCAACGATCTGAGAT‐3′
	Reverse	5′‐TTTGTGAAGACGGTTATGGTCAA‐3′
	Probe	5′‐TGAAACTCTTGCCTCGTCCACTCCG‐3′

Protein Encoded for, and Gene (Assay on Demand)	
18S ribosomal RNA (*18S FAST*)	Hs99999901_s1
Actin Beta (*ACTB*)	Hs99999903_m1
A disintegrin and metalloproteinase with thrombospondin motifs 4 (*ADAMTS4*)	Hs00192708_m1
A disintegrin and metalloproteinase with thrombospondin motifs 5 *ADAMTS5*)	Hs01095518_m1
Alkaline phosphatase (*ALPL*)	Hs00758162_m1
Chemokine (C‐C motif) ligand 18 (*CCL18*)	Hs00268113_m1
Cluster of Differentiation 163 (*CD163*)	Hs00174705_m1
Mannose Receptor C type 1 (*CD206*)	Hs00267207_m1
Interleukin 1 Beta *(IL1B*)	Hs00174097_m1
Interleukin 6 (*IL6*)	Hs00174131_m1
Interleukin 8 (*CXCL8*)	Hs00174103_m1
Interleukin 10 (*IL10*)	Hs00961622_m1
Nitric oxide synthase 2 (*NOS2*)	Hs01075529_m1
Piezo‐type mechanosensitive ion channel component 1 (*PIEZO1*)	Hs00207230_m1
Transcription factor SOX‐9 (*SOX9*)	Hs00165814_m1
Transient receptor potential cation channel subfamily V member 4 *(TRPV4)*	Hs01099348_m1
Tumor Necrosis Factor Alpha (*TNF*)	Hs00174128_m1

### Quantification of Sulfated Glycosaminoglycan per DNA (sGAG per DNA)

Pellets were lyzed in Proteinase K (PK, 0.5 mg mL^−1^) at 56 °C for 24 h, followed by inactivation at 95 °C for 10 min and storage at −20 °C upon further analysis. To quantify sulfonated glycosaminoglycan (sGAG), PK‐cell lysate was analyzed with 1,9‐dimethylmethylene blue (DMMB, pH 3) following previously published protocols.^[^ ^71^ ^]^ Absorbance was measured at 530 nm on a Synergy HTX multi‐mode plate reader (Biotek Instruments, Switzerland). DNA was quantified with Hoechst (33 258) in TNE buffer using DNA from the calf thymus as standard. Fluorescence intensity was measured at λ_ex_ = 485 nm and λ_ex _= 535 nm. Results are presented as median ± 95% CI for sGAG per pellet, DNA per pellet, and sGAG per DNA, from *n* = 3 pellets per condition from three individual experiments (three donors).

### Statistical Analysis

Sample sizes of each experiment are reported in the respective paragraphs. Statistical analysis was performed with GraphPad Prism (La Jolla, USA). Based on the small sample size of *n* < 30, the normal distribution of data points was assessed in a Shapiro‐Wilk test. Differences among the ranks of non‐normally distributed data for more than two groups were assessed in a non‐parametric Kruskal Wallis test, followed by pairwise comparison with Dunn's correction. Differences in the ranks between the two groups were assessed in a nonparametric Mann‐Whitney test. Significant differences were accepted for a P value < 0.05

## Conflict of Interest

The authors declare no conflict of interest.

## Supporting information



Supporting Information

## Data Availability

The data that support the findings of this study are available from the corresponding author upon reasonable request.
