# Supplementary material for: Conditioned Media from Mechanically Stimulated Macrophages Upregulate Osteogenic Genes in Human Mesenchymal Stromal Cells
Source: Adv Healthc Mater. 2025 Jun 22;14(31):2500706. doi: 10.1002/adhm.202500706 (PMC12683204; doi:10.1002/adhm.202500706)
Supplement: Supplementary file 1 — Supporting Information [file ADHM-14-0-s001.docx]

Supporting Information

**Conditioned media from mechanically stimulated macrophages upregulate osteogenic genes in human mesenchymal stromal cells**

Anne Géraldine Guex*, Ursula Menzel, Yann Ladner, Angela R. Armiento, Martin J. Stoddart*

Anne Géraldine Guex

AO Research Institute Davos, Davos, Switzerland

Department Research, University Center for Dental Medicine Basel UZB, University of Basel, Basel, Switzerland

Department of Biomedicine, University of Basel, Basel, Switzerland
E-mail: geraldine.guex@unibas.ch

Ursula Menzel
AO Research Institute Davos, Davos, Switzerland

Yann Ladner

AO Research Institute Davos, Davos, Switzerland

Institute for Biomechanics, ETH Zurich, Zurich, Switzerland

Angela R. Armiento

AO Research Institute Davos, Davos, Switzerland

Martin J Stoddart

AO Research Institute Davos, Davos, Switzerland

Department of Orthopedics and Trauma Surgery, Medical Center-Albert-Ludwigs-University of Freiburg, Faculty of Medicine, Freiburg, Germany

E-mail: martin.stoddart@aofoundation.org

*
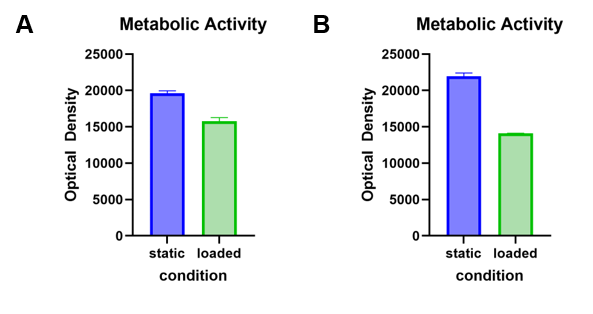
*

**Figure S1.** Cell metabolic activity. During initial screening experiments to evaluate and optimise parameters, cell metabolic activity of THP-1 cells in fibrin hydrogels was assessed with Cell Titer Blue. A) THP-1 were cultured for 3 days. Metabolic activity decreased in conditions subjected to mechanical stimulation compared to static conditions (80%) B) THP-1 were cultured for 3 days, applying an extended loading regime of 8 hours in total. Extensive loading resulted in reduced cell metabolic activity compared to the static control (64%). Based on these findings and experimental parameters established in our group by Fahy et al.,^[1]^ and Li et al.,^[2]^ a loading regime of 20% dynamic compression at 1 Hz shear was applied during 1 hour per day over 3 consecutive days.

[1] N. Fahy, U. Menzel, M. Alini, M.J. Stoddart, Shear and dynamic compression modulates the inflammatory phenotype of human monocytes in vitro, Frontiers in Immunology 10 (2019) 383.
DOI: <https://doi.org/10.3389/fimmu.2019.00383>.

[2] Z. Li, S.-J. Yao, M. Alini, M.J. Stoddart, Chondrogenesis of human bone marrow mesenchymal stem cells in fibrin–polyurethane composites is modulated by frequency and amplitude of dynamic compression and shear stress, Tissue Engineering Part A 16(2) (2009) 575-584.
DOI: <https://doi.org/10.1089/ten.tea.2009.0262>.


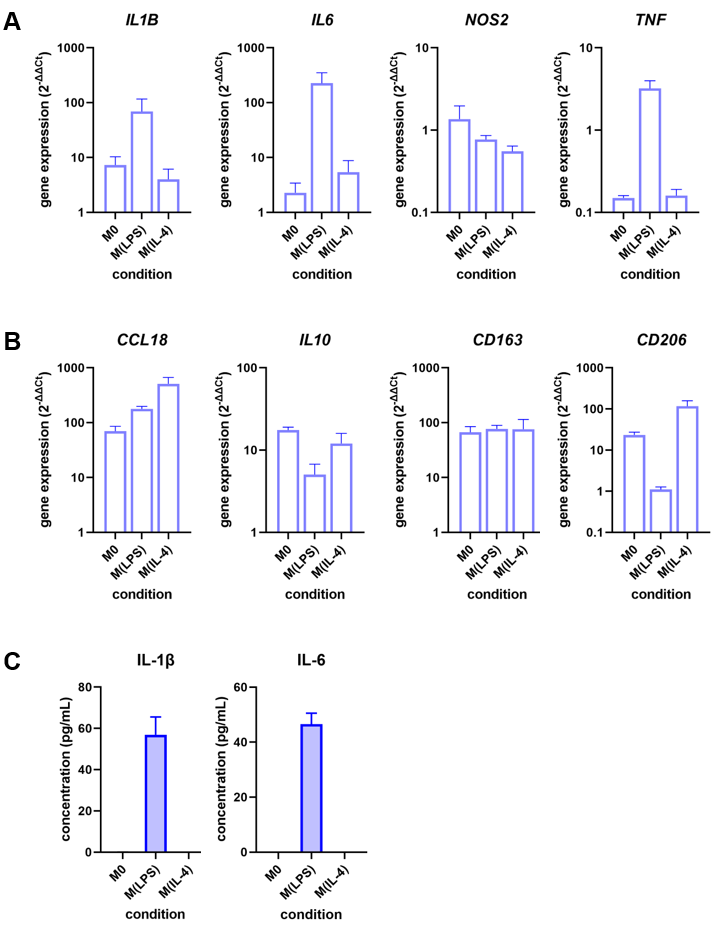


**Figure S2.** Gene expression of THP-1 macrophages cultured on 2-dimensional tissue culture treated poly(styrene) (TCPS). Monocyte to macrophage transition was induced with PMA for 24 hours, followed by a 72-h rest phase in complete RPMI medium. Following, polarisation was chemically induced during 48 h with LPS and IFNγ (M(LPS)) or IL-4 (M(IL-4)), respectively. Control conditions (M0) were cultured in complete RPMI medium. A) Genes encoding for pro-inflammatory proteins and B) anti-inflammatory proteins. In M(LPS), pro-inflammatory genes were upregulated compared to M0 or M(IL-4). Conversely, increased expression of CCL18, IL10, or CD206 was observed in M(IL-4) compared to the other two groups. Data are presented as median + 95% CI of one individual experiment with three wells per condition, normalised to the housekeeping gene 18S and macrophages harvested on day 0. C) Enzyme Linked Immunosorbent Assay (ELISA) for IL-1β, IL-6 or IL-10. Results for IL-1β or IL-6 confirm findings on the gene expression level. No IL-10 secretion was detected in any of the conditions. Data stem from N=1 individual experiment with n=3 biological repeats, no statistical analysis was performed.

*
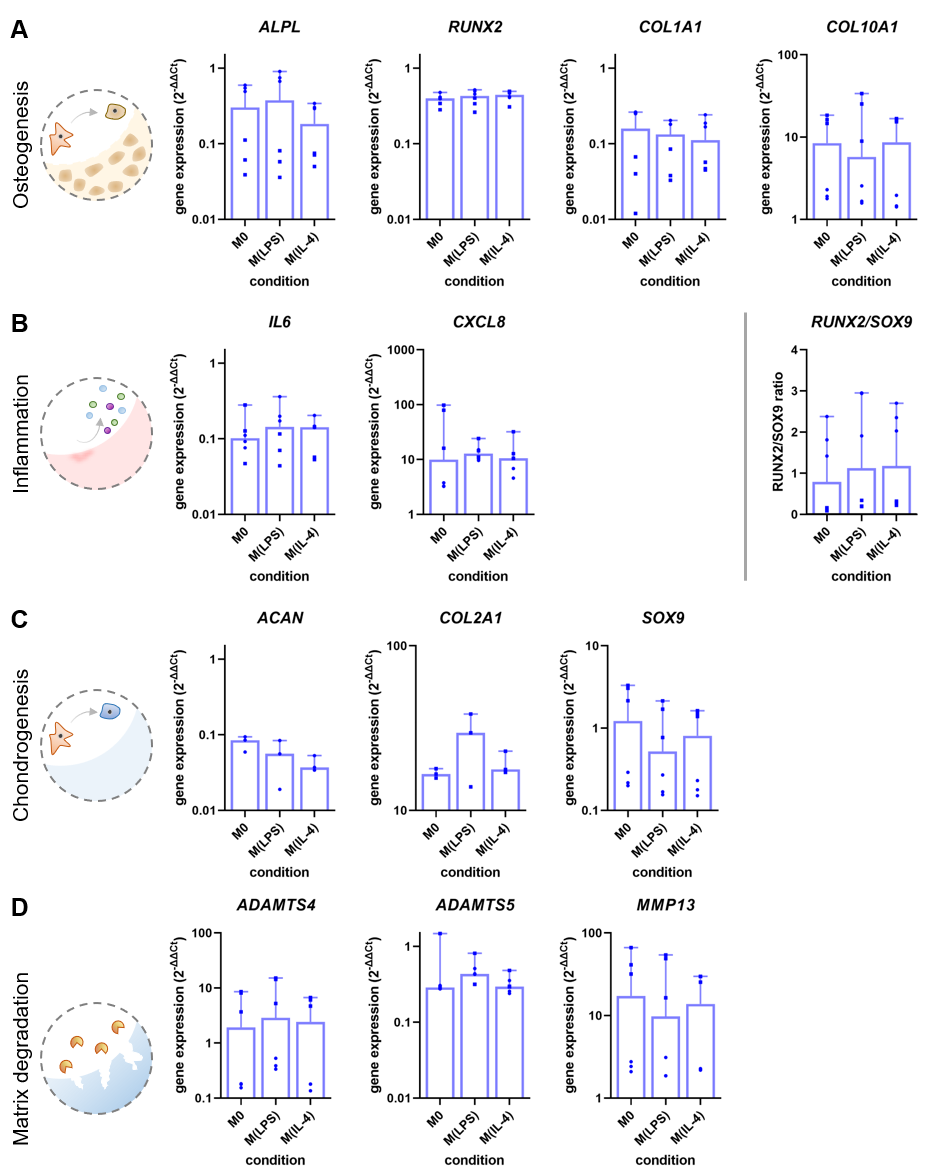
*

**Figure S3.** Gene expression profile of MSC cultured with conditioned media derived from macrophages on 2D (TCPS). M0, M(LPS) or M(IL-4) indicate the chemical stimulation. A) genes encoding for osteogenic markers, B) inflammatory proteins, C) chondrogenic markers, D) matrix degrading enzymes. Results are reported as median + 95% CI (bars and error bars). Individual datapoints are plotted as dots (donor 1) or rectangles (donor 2). Conditioned media of chemically polarised macrophages on 2D did not result in any significant changes in gene expression levels in MSC.


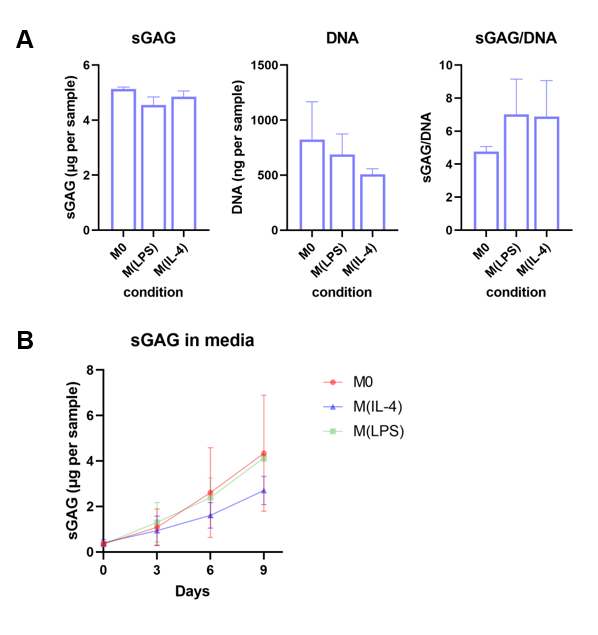


**Figure S4.** Extracellular matrix formation in MSC pellet cultures. A) sGAG, DNA or sGAG/DNA of MSC pellets after 9 days, cultured with conditioned media derived from macrophages cultured on 2D (TCPS). B) sGAG secreted into media. Results are presented as median + 95% CI of two individual experiments (donor 1 and donor 2) with one pellet per donor.

**
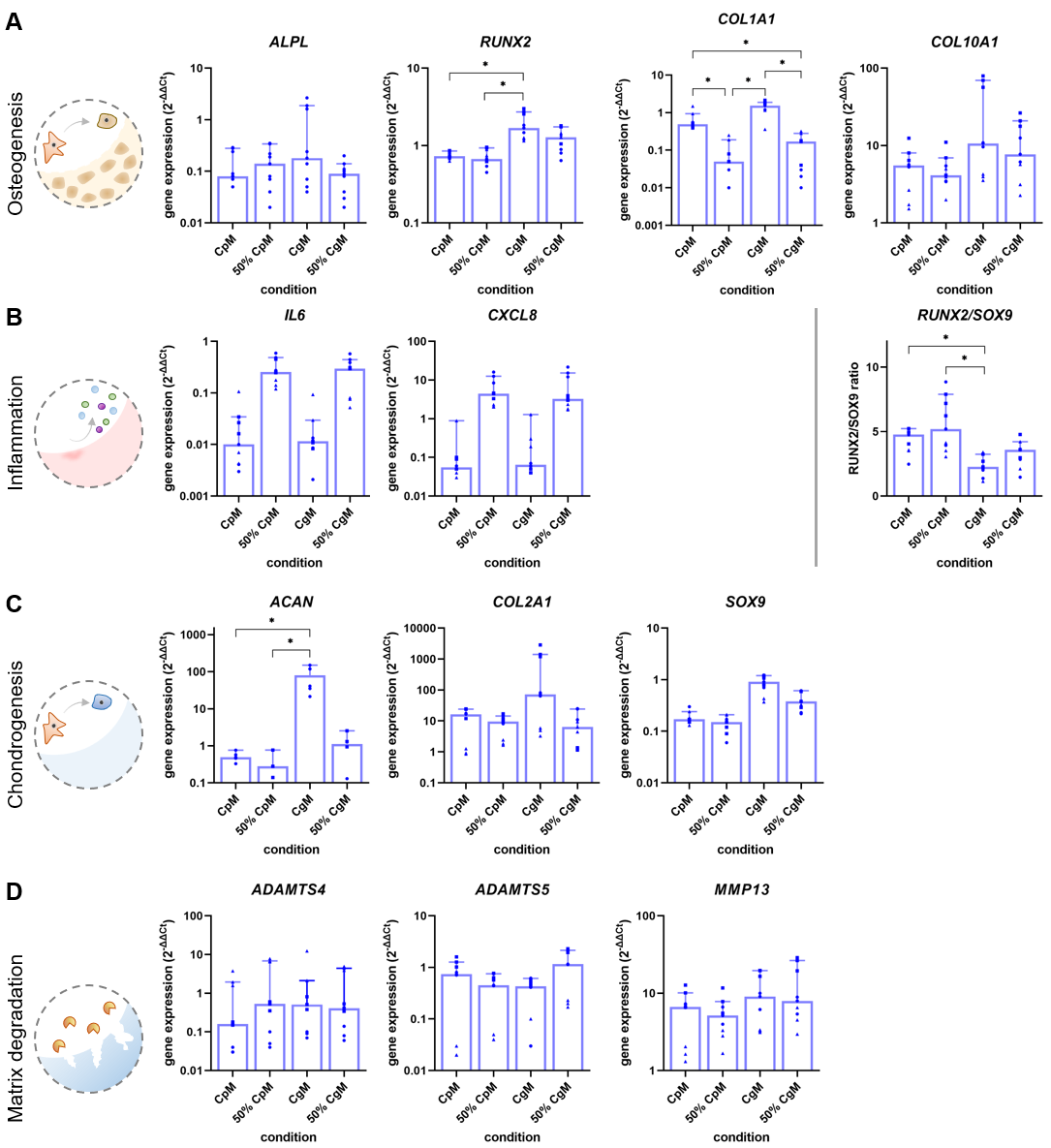
Figure S5.** Gene expression profile of MSC cultured under additional control conditions: with chondropermissive medium (CpM), chondrogenic medium (CgM, supplemented with 10 ng·mL^-1^ TGFβ_1_), or CpM or CgM mixed with complete RPMI medium at a 50:50 ratio. A) genes encoding for osteogenic markers, B) inflammatory proteins, C) chondrogenic markers, D) matrix degrading enzymes. Results are presented as median + 95% CI (bars and error bars). Individual datapoints are plotted as dots (donor 1), rectangles (donor 2), or triangles (donor 3) and normalised to the housekeeping gene RPLP0 and MSC pellets harvested after 24 h (2^-Δ∆Ct^). *p<0.05.

*
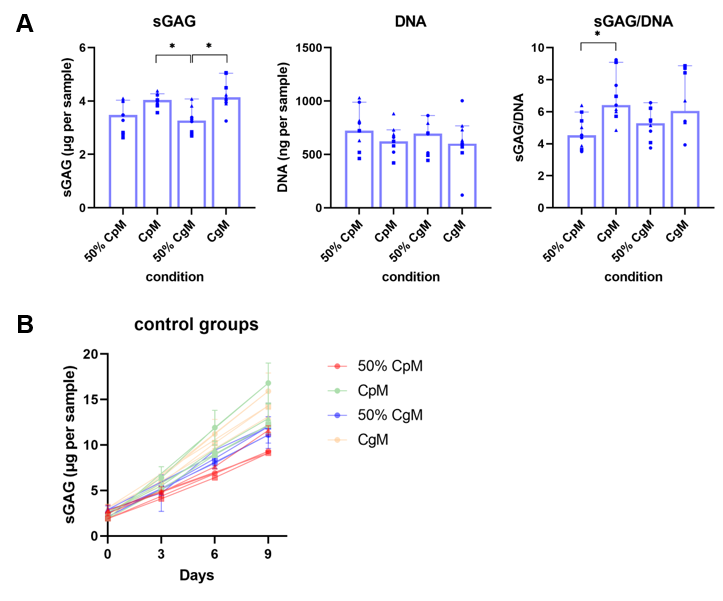
*

**Figure S6.** Extracellular matrix formation in MSC pellet cultures. A) sGAG, DNA or sGAG/DNA of MSC pellets after 9 days, cultured with chondropermissive medium (CpM), chondrogenic medium (CgM, supplemented with 10 ng·mL^-1^ TGFβ), or CpM or CgM mixed with complete RPMI medium at a 50:50 ratio. Results are presented as median + 95% CI (bars and error bars). Individual datapoints are plotted as dots (donor 1), rectangles (donor 2) or triangels (donor 3). *p<0.05. B) sGAG secreted into media. Results are presented as median ± 95 CI of three individual samples per donor per condition.
